# Meteorological Observations for February, 1857, Atlanta, Ga.

**Published:** 1857-03

**Authors:** J. G. Westmoreland


### METEOROLOGICAL OBSERVATIONS FOR FEBRUARY, 1857, AT ATLANTA, GA.

| FEBRUARY. | TEMPERATURE. | | | WIND. | REMARKS. |
|---|---|---|---|---|---|
| | 7 A. M. | 2 P. M. | 7 P. M. | | |
| 1 | 35 | 54 | 40 | N. W. | Fair. |
| 2 | 34 | 48 | 40 | W. | Fair. |
| 3 | 38 | 60 | 54 | S. E. | Fair. |
| 4 | 40 | 66 | 50 | N. E. | Fair. |
| 5 | 52 | 63 | 56 | S. | Cloudy. |
| 6 | 52 | 67 | 59 | S. W. | Cloudy. |
| 7 | 56 | 75 | 59 | W. | Hazy. |
| 8 | 50 | 44 | 30 | S. W. | Cloudy—Rain ½. |
| 9 | 22 | 46 | 35 | N. W. | Fair. |
| 10 | 24 | 46 | 32 | N. W. | Fair. |
| 11 | 26 | 40 | 36 | W. | Fair. |
| 12 | 32 | 50 | 44 | S. E. | Light clouds—Drizzily. |
| 13 | 40 | 54 | 48 | S. E. | Hazy. |
| 14 | 46 | 62 | 52 | S. | Hazy. |
| 15 | 48 | 65 | 60 | S. W. | Fair. |
| 16 | 54 | 68 | 58 | S. W. | Rain 1-16. |
| 17 | 54 | 70 | 64 | W. | Fair. |
| 18 | 50 | 76 | 52 | N. E. | Fair. |
| 19 | 52 | 84 | 62 | S. W. | Fair. |
| 20 | 56 | 78 | 60 | S. | Hazy. |
| 21 | 58 | 66 | 58 | S. | Hazy. |
| 22 | 40 | 66 | 52 | W. | Hazy. |
| 23 | 40 | 70 | 56 | W. | Fair. |
| 24 | 53 | 74 | 62 | S. W. | Hazy. |
| 25 | 62 | 76 | 62 | W. | Hazy. |
| 26 | 52 | 76 | 56 | W. | Fair. |
| 27 | 54 | 80 | 64 | W. | Fair. |
| 28 | 52 | 66 | 46 | W. | Drizzily. |

Furnished by

J. G. WESTMORELAND, M. D.

